# Sequence-Only Prediction of Super-Enhancers in Human Cell Lines Using Transformer Models

**DOI:** 10.3390/biology14020172

**Published:** 2025-02-07

**Authors:** Ekaterina V. Kravchuk, German A. Ashniev, Marina G. Gladkova, Alexey V. Orlov, Zoia G. Zaitseva, Juri A. Malkerov, Natalia N. Orlova

**Affiliations:** 1 Prokhorov General Physics Institute of the Russian Academy of Sciences, 38 Vavilov St., 119991 Moscow, Russia; kravchuk_ekaterina01@mail.ru (E.V.K.); ashnievh@gmail.com (G.A.A.); gladkovamg@my.msu.ru (M.G.G.); zajzoya@yandex.ru (Z.G.Z.); jurimalkerov@yandex.ru (J.A.M.); 2 Faculty of Biology, Lomonosov Moscow State University, Leninskiye Gory, MSU, 1-12, 119991 Moscow, Russia; 3 Institute for Information Transmission Problems RAS, 127051 Moscow, Russia; 4 Faculty of Bioengineering and Bioinformatics, Lomonosov Moscow State University, GSP-1, Leninskiye Gory, MSU, 1-73, 119234 Moscow, Russia

**Keywords:** bioinformatics, super-enhancers, neural networks, transformers, BigBird, BERT

## Abstract

The study discloses the application of transformer-based deep learning models for the task of super-enhancers prediction in human tumor cell lines with a specific focus on sequence-only features within studied entities of super-enhancer and enhancer elements in the human genome. The proposed SE-prediction method included the GENA-LM application at handling long DNA sequences with the classification task, distinguishing super-enhancers from enhancers using H3K36me, H3K4me1, H3K4me3 and H3K27ac landscape datasets from HeLa, HEK293, H2171, Jurkat, K562, MM1S and U87 cell lines. The model was fine-tuned on relevant sequence data, allowing for the analysis of extended genomic sequences without the need for epigenetic markers as proposed in early approaches. The study achieved balanced accuracy metrics, surpassing previous models like SENet, particularly in HEK293 and K562 cell lines. Also, it was shown that super-enhancers frequently co-localize with epigenetic marks such as H3K4me3 and H3K27ac. Therefore, the attention mechanism of the model provided insights into the sequence features contributing to SE classification, indicating a correlation between sequence-only features and mentioned epigenetic landscapes. These findings support the potential transformer models use in further genomic sequence analysis for bioinformatics applications in enhancer/super-enhancer characterization and gene regulation studies.

## Simple Summary

This research focuses on advanced transformer models application for super-enhancers prediction in human cell lines purely from DNA sequence data. By applying the GENA-LM transformer model, effective differentiation between super-enhancers and enhancers across multiple cell lines was achieved compared to previously used techniques. The approach suggests sequences processing without any additional information about epigenetic landscape within fine-tuned model application for the enhancer/super-enhancer classification task.

## 1. Introduction

In 2013, the term “super-enhancer” (SE) was proposed as a new class of regulatory elements. Generally speaking, super-enhancers represent a superclass (multiple clusters) of dynamic enhancer elements showing atypically high enrichment in transcription factors binding represented in a wide cohort of different cell types (thus including both undifferentiated and differentiated cells). The definition of SE is still unclear but usually includes a list of characteristic features that correlate with the presence of active super-enhancers in various cell lines. Most in vitro studies highlight the local enrichment of transcription factors (TFs) such as BRD4, Mediator 1 (Med1), MyoD, etc., as well as epigenetic marks (H3K4me1, H3K27ac, H3K4me3 along with cohesin and CTCF binding) and specific spacing between enhancer elements (max = 12.5 kb) [1,2]. It has been shown that these elements play an important role in the development of various diseases, including different types of cancer [3], in addition to the processes key to cell biology, determining the functioning of stem cells and differentiation.

The initial approaches for SEs search were to determine the level of Med1 binding as measured by chromatin immunoprecipitation and sequencing (ChIP-seq). Whyte’s definition included the following features [1]: (i) enhancers are chromatin sites that bind to all three master regulators, Oct4, Sox2, and Nanog, according to ChIP-seq data; (ii) enhancers united within 12.5 kb are defined as super-enhancers; (iii) super-enhancers used to have high relative rank by the normalized Med1 signal level while stretched enhancers (StEs) and the remaining typical enhancers (TEs) revealed low Med1 signals.

The challenge of predicting super-enhancers began with traditional machine learning (ML) techniques, among which the linear rank-ordering of super-enhancers (ROSE) algorithm has become the gold standard [1,4]. In the ROSE framework, super-enhancers are grouped based on a specified threshold and ranked according to the increasing total background-subtracted Med1 ChIP-seq signal. Thus, the specified threshold for the top-ranked entity segmentation divides super-enhancers from stretched and typical enhancers.

Some authors deviate from this definition by including different (single and combined) labels for the deeper characterization of SEs with or without the use of the ROSE algorithm. For example, certain studies have exclusively utilized H3K27ac to identify super-enhancer regions [3], while other groups have primarily relied on the Med1 signal [4]. Thus, the only common and therefore clearly defining feature of super-enhancers is their exceptionally high enrichment in transcriptional activators and chromatin marks, as determined through ChIP-seq, which aligns with the original third SE feature proposed by Whyte.

Another ML-based analysis was conducted to assess the significance of various potential super-enhancer (SE) features, leading to the development of the imPROSE (integrated methods for the prediction of SEs) tool [5]. Integrative data from ChIP-seq, RNA-seq, gene ontology, and DNA motifs were used in six classic machine learning models proposed: Decision Tree, Random Forest, linear support vector machine (SVM), k-Nearest Neighbors (k-NN), adaptive boosting (AdaBoost), and Naive Bayes. Through a 10-fold cross-validation process and a comparison of the different methods, it was determined that the Random Forest model yielded the best performance, achieving an AUC of 0.98. When SEs were characterized based on DNA sequence features (phastCon, GC content, and repeat fraction scores), the authors estimated a comparable AUC of 0.81. This led to the hypothesis that DNA characteristics (nucleotide context, conservation, specific mutations etc.) alone could effectively classify typical enhancers (TEs) and super-enhancers (SEs).

Deep learning technologies were later used for SE research with several works offering Convolutional Neural Networks solutions. The first AI model used for this purpose DEEPSEN, implemented in 2019, utilizing ChIP-seq and DNase-seq datasets [6]. By 2021, a new algorithm called DeepSE was published, which focused on sequence feature embeddings only [7]. This approach aims to classify typical enhancers and super-enhancers in two classes using k-mer sequence embeddings generated by training dna2vec on genomic data along with a CNN classifier that includes two convolutional layers and two fully connected layers for binary classification. Despite relatively high accuracy values, the precision metric of DeepSE did not exceed 0.48 and the F1 score did not exceed 0.52, which resulted in the tool’s limited popularity.

For similar DNA sequence classification tasks, such as typical enhancer annotation, Convolutional Neural Networks (CNNs), recurrent neural networks (RNNs), hybrid models [6], and transformers were employed. An example of such a hybrid architecture, BiRen, was introduced in 2017. BiRen integrates the sequence encoding and representation power of a CNN with the superior capacity of gated recurrent unit (GRU)-based bidirectional recurrent neural network (BRNN) to manage long-term dependencies in extended DNA sequences, allowing the accurate identification of enhancers using the DNA sequence alone [7].

For the same enhancer prediction task using only the DNA sequence as input, the DNABERT model—a Bidirectional Encoder Representations from Transformers (BERT)-based transformer neural network architecture—was developed [8]. A pre-trained version of DNABERT is available, which can be utilized for various analyses of non-protein-coding genetic elements, including the annotation of enhancers and super-enhancers [9]. On the contrary, some researchers prefer to solely rely on ChIP-seq data for TE prediction, which is addressed by a hybrid model that combines CNNs and RNNs, using histone modification marks as input [10].

Published in 2023, the SENet algorithm aims at SE prediction solely based on sequence information within short genomic contexts [11]. The authors utilized a customized dataset obtained from the SEdb 2.0 database, which was filtered for sequences of 3000 bp in length and interspersed with reference mouse and human genomes. The pipeline included dna2vec feature embedding for sequence information representation; CNN layer for local feature extraction; attention pooling for refined feature retention and a transformer layer for extracting and refining contextual information.

GENA-LM (BigBird-base T2T) [9] is a transformer masked language model trained on human DNA sequences, which follows BigBird architecture [12]. BigBird is a sparse-attention based transformer which extends transformer-based models (such as BERT) to handle much longer sequences. It employs global and random attention mechanisms, allowing it to approximate full attention while requiring fewer computational resources [12]. Unlike DNABERT, GENA-LM uses Byte-Pair Encoding (BPE) tokenization instead of k-mers, supports an input sequence size of approximately 24,000 bp compared to DNABERT’s 510 bp, and is pre-trained on the T2T vs. GRCh38.p13 human genome assembly.

In our work, we tried to focus on predicting human super-enhancers. To achieve this, we selected publicly available datasets from various human cell lines of different origins, including HeLa, HEK293, H2171, Jurkat, K562, MM1S, and U87. We adapted the GENA-LM algorithm’s pipeline by preprocessing the input data, specifically removing enhancer regions with potentially ambiguous classifications to clean the publicly available data from the noise of typical enhancers, and conducting custom fine-tuning with further TEs and SEs classification and interpretation for HEK293 and K562 cell lines.

## 2. Materials and Methods

### 2.1. Software Implementation

All computations were performed using Python 3 in Google Colab with Google Cloud server resources, as well as utilizing Yandex Cloud services. The following libraries and frameworks were utilized: pybedtools and fuc for data preprocessing; PyTorch, Scikit-learn, Datasets, Transformers, wandb, tqdm, and random for model fine-tuning; Captum for interpretability analysis; and pandas, numpy, and matplotlib for data processing and visualization. The code is publicly available on GitHub (https://github.com/Kravchuk-Ekaterina/GENA-LM-SE) (accessed on 12 December 2024).

### 2.2. Datasets

Using open databases (dbSUPER [13], SEdb [14], EnchancerAtlas 2.0 [15], Encode (ENCODE portal, arxiv; PMID: 31713622; and PMCID: PMC7061942), we collected 7 datasets for different cell lines (Table 1). The datasets contain the same number of samples of enhancers and super-enhancers. To create balanced datasets that have the same distribution of length in tokens, we split both datasets into sequence bins sorted by lengths in tokens, which were 50 tokens apart. The number of sequences in each bin for SE and TE was chosen to be the smallest for these two sets of data, while randomly, part of the sequences from the dataset for which there were more of them in this bin was excluded.

The data from the resulting dataset is filtered so that the sequences in the dataset do not overlap with each other with coding and other regulatory sequences. In addition, super-enhancers shorter than 2500 bp were also filtered out. We set an upper length threshold of 25,000 bp, and sequences longer were trimmed.

To avoid data leakage, we approximated the initial length distribution of enhancers to the length distribution of super-enhancers by supplementing the enhancer sequences with flanking sequences. This procedure can be described as follows:

The SE length distribution is divided into *M* bins with the step of 50 bp. TE length distribution is then divided into the same amount (*M*) of bins. The intervals are numbered, and iteration occurs. A sequence with the length (*l*) is selected from the distribution of typical enhancer lengths from an interval with a certain number. It has a start coordinate (*s*) and the end coordinate (*e*). A random number *N* is selected from the interval with the same number of SE length distribution. This number *new_l* is the new length for the TE sequence. TE obtains a new start coordinate *new_s*, which is a random number between *s—(new_l—l)* and *s*. The new end coordinate is *new_s + new_l*.

Using the coordinates in the obtained dataset, we generated sequences using MEME Suite [16] and the assembly of the hg19 human genome. The datasets with sequences were used further.

Next, to minimize noise in flanking sequence sets and improve model training efficiency, we filtered sequences using human genome annotation obtained from the UCSC Browser within each cell line. As a result, the class of training set corresponding to enhancer elements was formed so that open reading frames (ORFs) and other non-contextual groups of nucleotides in the flanking regions of the sequences served as exclusion criteria. Thus, we avoided overfitting due to the presence of other regulatory elements or coding sequences in the dataset.

**Figure 1:**
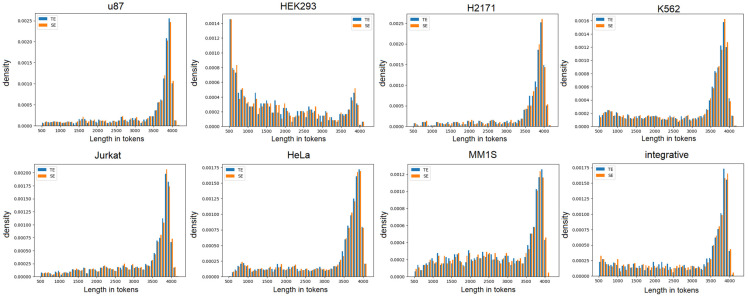
The distribution of length of the sequences in tokens for datasets for different cell lines.

To create an integrative dataset, we mixed other datasets and randomly chose 2500 sequences from this mixed data. Since the task of binary classification was being solved, the dataset contained class labels: 0 (TE) and 1 (SE). We split the data randomly into train and test datasets in a ratio of 3:1 and performed BPE tokenization (more details in Section 2.3.1). We selected the sequences in such a way that the distribution of length in tokens for TE and SE were similar (Figure 1).

Peaks of the distributions are shifted toward longer lengths are between 500 and 4000 tokens, which suggests that most super-enhancers exceed 20,000 nucleotides in length, which once again emphasizes the importance of analyzing long sequences in the context of the problem under consideration. All the length distributions look similar except for the one for the HEK293 cell line. This can be explained by the fact that for this cell line, all data were obtained from SEdb, while for others, they were obtained from SEdb and dbSuper (Table 1).

Notably, during selection of the fixed cell lines dataset, we balanced between SeDB 2.0 data availability and representation diversity of cancer types rather than focusing on a specific family of cells or tissue related to one organ or system. This approach allowed for a more comprehensive understanding of SE-driven cancer biology. Despite our technically narrowed view for various cell lines and tissues, the inclusion of hematological cell lines alongside solid tumor models could help to investigate shared mechanisms that may exist between different cancer types. Additionally, selecting a diverse set of cell lines helped to prevent potential model overfitting, as the super-enhancer prediction algorithm operates solely on DNA sequence data. Restricting the dataset to a single disease or closely related cell types could bias the model toward detecting super-enhancer elements only in specific contexts. Finally, we also considered the availability of the selected cell lines to facilitate potential future experimental validation.

HEK-293 cells, originally derived from human embryonic kidney cells through adenovirus-mediated transformation, exhibit tumorigenic properties, including rapid proliferation and suppressed cell-cycle checkpoints. These characteristics make them a relevant model for studying cancer cell behavior. Despite their embryonic origin, HEK-293 cells share several key features with cancerous cells, allowing for meaningful comparisons in the context of super-enhancer regulation and tumorigenesis.

### 2.3. Methods

#### 2.3.1. The Model Used for SE Prediction

**Figure 2:**
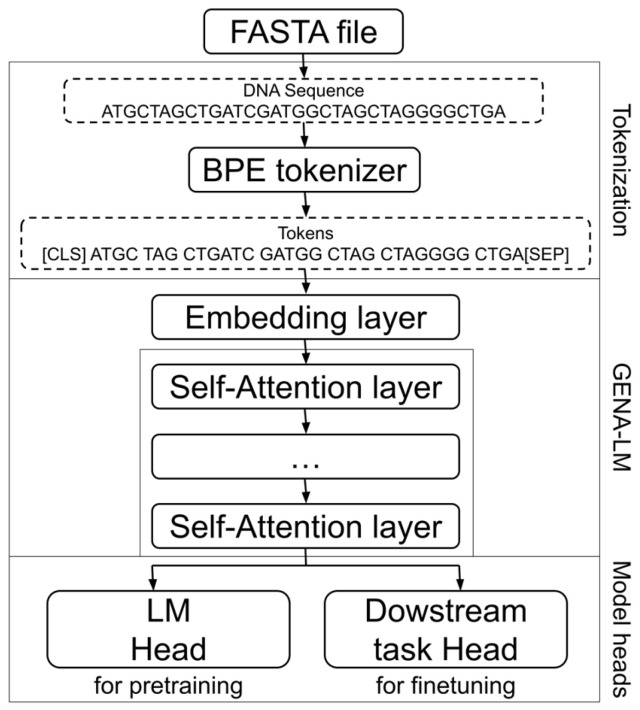
The GENA-LM transformer-based architecture diagram based on [9].

We performed fine tuning of the GENA-LM BigBird model (specifically, the gena-lm-bigbird-base-2t2 variant) to enhance its performance on obtained data. The architecture of this model is illustrated in Figure 2, while a more detailed architectural diagram can be found in Figure S1. The GENA-LM (gena-lm-bigbird-base-t2t) model was trained using a masked language modelling (MLM) approach, as proposed in the BigBird paper [12]. This involved masking 15% of the tokens in the input sequences, allowing the model to learn contextual relationships and dependencies within the data. The configuration of the GENA-LM model is similar to the google/bigbird-roberta-base, featuring the following specifications:

The model can process sequences up to 4096 tokens, which corresponds to approximately 36,000 base pairs. It consists of 12 layers and utilizes 12 attention heads. The hidden size of the model is set to 768, determining the dimensionality of the embeddings used within the transformer architecture.

In terms of the sparse attention mechanism, the model utilizes a block size of 64, allowing for effective memory and computational resource management while processing long sequences. The architecture includes 3 random blocks, where tokens can attend to a randomly selected subset of other tokens, facilitating the capture of long-range dependencies. Additionally, there are 2 global blocks that allow certain tokens to attend to all other tokens in the sequence, ensuring that critical information is not lost in the attention mechanism. The model also includes 3 sliding window blocks, which enable local attention patterns, allowing tokens to focus on their closest neighbors.

The input to the model is nucleotide sequences from the fasta file, which go through BPE tokenization. The GENA-LM model includes embedding layers and self-attention layers. Binary classification is performed by a fully connected model head (Figure 2).

Byte-Pair Encoding (BPE) [17,18] is a tokenization algorithm that relies on a pre-tokenizer that splits the training data into words. After pre-tokenization, a set of unique words is created, and the frequency with which each word occurs in the training data is determined. The algorithm then creates a basic dictionary containing all the characters appearing in the set of unique words and corresponding to the character occurrence in a given word, from which it learns, merging rules to form a new character from two characters in the basic dictionary. So, at the beginning, these merges will create tokens with two characters, and then, as training progresses, they will create longer subwords. The process repeats until the vocabulary reaches the target size. The target dictionary size is a hyperparameter determined before training the tokenizer. In the case of GENA-LM, the vocabulary size is 32,000, and BPE tokenizer was trained on DNA data (using T2T-CHM13v1.1 genome assembly).

**Figure 3:**
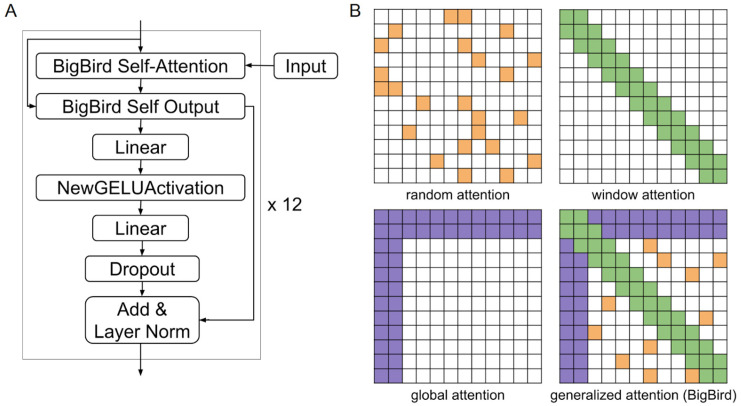
BigBird attention. (**A**) BigBird self-attention layer, (**B**) building blocks of the random attention (yellow ochre), window attention (green), global attention (violet) and generalized attention (adapted from [12]).

The embedding layer is the first layer of transformers, including BigBird. If we take a single length *N* sequence of *D_in_*-dimensional vectors, *X ∈ R^N × Din^*, each vector can be transformed into a vector of size *D_out_*. In language tasks, the input is a sequence of tokens, *D_in_* = 1. The transformation can be linear or more complex: for example, another neural network. The output of this procedure is an embedded sequence shaped (*N*, *D_out_*). Then, a positional encoding is added to the embedding to contain positional information for each vector of the sequence. These encodings also can be fixed or learned [19]. BigBird is a model with absolute position embeddings. The model has 12 self-attention layers and 12 attention heads [12]. The diagram of the BigBird self-attention layer is represented in Figure 3A and Figure S1. The model uses a special attention mechanism—generalized attention. It integrates qualities of random attention, sliding window attention and global attention mechanisms (Figure 3B). BigBird’s attention mechanism is computationally very efficient for long sequences.

The classification head includes the following: dropout, linear layer followed by NewGELUActivation, dropout, linear layer (Figure S1). The output shape is (1, 2), since the model solves the problem of binary classification. Fine tuning was performed first for the classifier (5 epochs) and then for all layers of the model (10 more epochs). The key information about the hyperparameters is contained in Table S1. The used loss function is cross-entropy loss.

#### 2.3.2. Evaluation Metrics

In this study, we used classical metrics to assess the performance of the pretrained GENA-LM model (Table S2).

#### 2.3.3. Interpretation of the Results

To interpret the results, we used the captum package (https://captum.ai/) (accessed on 20 January 2025) to set the layer conductance [20,21] and use the integrated gradients algorithm [22,23]. The integrated gradient algorithm helps to solve the problem of attributing the prediction of a neural network to its input features, which is of particular interest to us. This information gives us insight into the connection with biological characteristics. We calculated the attribution sum for 5 SEs top-ranked by the model that were from test datasets for each cell line and converted these scores to scores in a .bed file. These captum scores reflect the contribution of the token to the model output. Tokens with positive scores increase the probability of assigning a sequence to class 1, negative ones to class 0. For ease of visualization in the genome browser, scores were multiplied by a factor equal to 1000/(maximum absolute value of scores). We made a visualization in the UCSC genome browser [24] with the observed scores in grayscale (the highest values are darker). We divided the scores into positive (which are considered to be presented in SE class) and negative (scores for common features for TE class).

We compared captum results in the UCSC browser with some other features, such as epigenetic marks, ChIP-Atlas and ReMap ChIP-seq data [1,3,4,25,26]. Our goal was to see how the captum results correspond to this experimental data.

## 3. Results and Discussion

### 3.1. GENA-LM Fine-Tuning for Solving the Problem of Predicting Super Enhancers

Here, we performed two steps of fine-tuning for the GENA-LM model: first fine-tuning of the classifier and the second fine-tuning of the full model.

**Figure 4:**
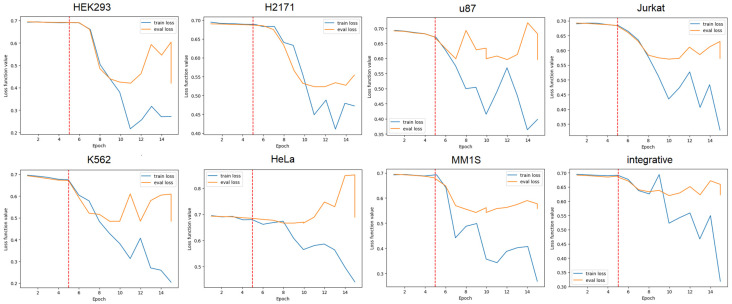
Changes in the loss function values during training; the red line is the epoch when fine tuning of all layers of the model begins.

As the training progressed, we observed a decrease in both the training loss and the validation loss for both fine-tuning stages. The most significant decrease was observed on the full model training step (Figure 4 and Figure S2). Based on the loss curves nature, we determined that 10 epochs were sufficient for training. More epochs have led to signs of model overfitting.

**Figure 5:**
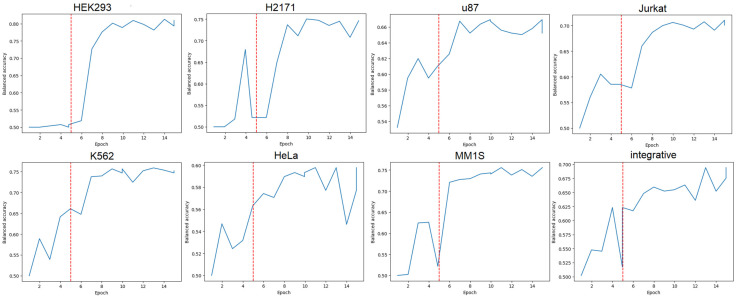
Changes of accuracy during training; the red line is the epoch when fine tuning of all layers of the model begins.

Classification metrics, such as balanced accuracy, increased on both stages (Figure 5 and Figure S2, Table 2). The obtained results show that the transformer, pretrained on human genomic data, is capable of extracting the information from the DNA sequence to distinguish such close elements as SE and TE. Also, the t-SNE and UMAP visualizations of the feature space distribution of the output of the GENA-LM transformer layer show the separation for these two classes for all datasets (Figures S3 and S4).

The obtained metrics are presented in Table 2. The accuracy score outperforms 0.7 for most of the datasets, reaching 0.81 for HEK293.

We performed McNemar’s test to compare the fine-tuned model with a random classifier. For all the cell lines, the difference between GENA-LM and random classifier is significant (*p*-value < 0.05) (Table S3).

For a number of cell lines (HEK293, H2171, K562, MM1S), accuracy is higher than for previously published SENet trained and tested on human data, which is 0.74 [11]. Other metrics considered are also higher for these cell lines and comparable for others. The integrative model shows accuracy 0.69, which is comparable to the results of SENet.

In addition, we can compare the results for H2171, u87, the MM1S of our model and DeepSE [27]. In the case of DeepSE, although the accuracy values were slightly higher than those of GENA-LM, DeepSE has low precision (0.39 for H2171, 0.17 for u87, 0.32 for MM1S) and f1 metrics (0.31 for H2171, 0.52 for u87, 0.46 for MM1S). Low precision in the case of DeepSE demonstrates that more than half of the sequences that are considered super enhancers by this model are not actually super enhancers for all the cell lines considered. In the case of GENA-LM, the values of these metrics are comparatively high.

Next, we decided to fine-tune the model on the integrative dataset, which had the same number of samples for each cell line, and test the results of this model on various test datasets. We took 800 sequences from each cell line dataset, as this number is comparable to the size of the smallest dataset, pooled them, and randomly selected 2500 sequences to fine-tune the integrative model. This was performed to avoid biases due to differences in the dataset sizes for each cell line.

The metric values were lower than in the case of models fine-tuned on data for each cell line (Table 3), which may be due to the tissue specificity of super-enhancers. Also according to McNemar’s test, which compares the fine-tuned model with a random classifier, there is a significant difference between GENA-LM and random classifier (*p*-value < 0.05) only for HEK293 and Jurkat (Table S3).

To validate the specificity of the model’s predictions, we included negative controls. We created a dataset with 50 random sequences from the human genome with random lengths from 2500 to 5000 bp and performed the Kolmogorov–Smirnov test to compare the performance of all the fine-tuned models with the performance of the random classifier. As a result, we found no significant difference between GENA-LM and a random classifier (Table S4), as expected, since this dataset did not contain representatives of any of the classes on which the models were trained.

The idea that super-enhancers are not only tissue-specific but also play an important role in cell differentiation is consistent with the literature [1,3]. We speculate that the integrative model performed worse than the cell lineage-tuned models for this reason. The model did not generalize the overall differences between super-enhancers and typical enhancers as well as it did for individual cell lines, which was possibly due to a lack of data. For this reason, in the following, we decided to focus on the results for individual cell lines.

Like other models that make predictions based on sequence data alone, GENA-LM underperforms models that also use other features, such as ChiP-seq data. However, when compared to SENet and DeepSE, which solve the same problem, GENA-LM has several advantages. The use of GENA-LM allowed us to consider longer sequences, since super-enhancers are extended regulatory elements. Therefore, the model can be used for extended super-enhancers, and there is no need to filter them out due to technical limitations. It becomes possible to obtain results on different cell lines using a transformer, which had not been accomplished before.

### 3.2. Interpretation of Fine-Tuned Model

In this section, we describe the application of the GENA-LM deep learning model for recognizing super-enhancers with a focus on the genomes of the HEK293 and K562 cell lines. The selection of HEK293 and K562 cell lines for GENA-LM model application was based on their superior signal-to-noise ratios in super-enhancer and epigenetic mark visualization. While MM1S ranked second in model performance, HEK293 and K562 were prioritized due to their relevance in gene regulation studies and the overall quality of data obtained. Ensuring high-quality input data was critical for the robustness of downstream analysis, making these cell lines the most suitable choices for detailed evaluation. We decided to use these cell lines to provide initial insights into algorithm-based predictions using genomic context only. Related tissues and cell lines have already been experimentally analyzed by other research groups during various research tasks. Our goal here was to demonstrate the generality of the computational approach and to perform cross-validation of predictions with known distributions of regulatory elements. Additionally, we are committed to conducting experimental verification of the predicted loci, which imposes further limitations regarding the characteristics of the initial datasets.

GENA-LM generated attention subsequences within the top-five predicted super-enhancers for each cell line, and each attention subsequence was assigned a significance value, which was represented as a distribution of dark-orange-colored scores. Current visualization also includes the distribution of epigenetic marks such as H3K4me3, H3K4me1, H3K27ac, and H3K36me3 for each selected SE from HEK293 and K562 cell lines. The selection of four epigenetic marks relies on their association with an active chromatin state and elevated expression of enhancer or super-enhancer target genes.

H3K4me3 is historically associated with active promoters and is often found at the transcription start sites of actively expressed genes [28]. H3K4me3 is believed to be involved in the recruitment of transcriptional machinery and the initiation of gene transcription; however, recent studies question its role in transcription initiation and describe H3K4me3 mark as a transcriptional pause–release and elongation response [29]; meanwhile, H3K4me1 is associated with enhancer regions, featuring both active and deactivated enhancers [30]. H3K4me1 was reported as an initial activator mark, which can then be further co-activated by the addition of other histone modifications, such as H3K27ac [31,32]. H3K27ac is an indicator of active enhancers and promoters. It facilitates the recruitment of transcriptional regulators and promoters to loci characterized by an open chromatin state, thereby enabling an active transcription of target genes [33]. H3K36me3 is associated with the transcribed regions of actively expressed genes and regulates the process of transcription elongation [34] as well as takes part in the processing of nascent RNA transcripts [35].

**Figure 6:**
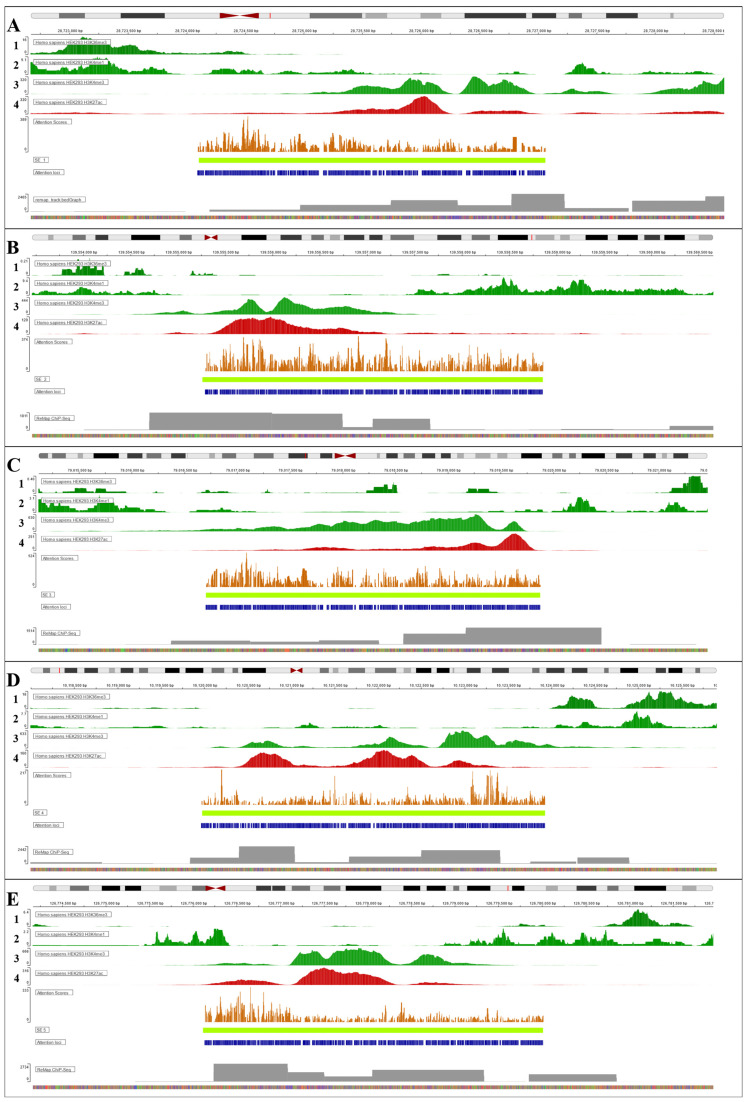
Visualization of intersection between epigenetic marks (dark green and red) and model attention positive class subsequences (blue) for 5 SEs (**A**–**E**; light green) from the HEK293 cell line. Epigenetic marks are numerated as follows: 1. H3K36me3, 2. H3K4me1, 3. H3K4me3, 4. H3K27ac. Values of attention score for each positive class subsequence represented as diagram (orange). ReMap Atlas track of regulatory regions is shown as a gray distribution pattern and is based on a comprehensive integrative analysis of all publicly available ChIP-seq data. Visualized in Integrative Genomics Viewer.

For the HEK293 cell line (Figure 6), the SEs showed a weak concordance between the importance evaluation of positive subsequences and ChIP-Atlas peaks visualization, indicating that the model’s importance scores did not strongly align with the integrative signal of epigenetic marks in this cell line. Nevertheless, each super-enhancer included both H3K4me3 and H3K27ac histone modification marks with almost a full absence of H3K4me1 and H3K36me3 signals. The locus of SE on Figure 6A (chr17 q11.2) contains the *Homo sapiens* carboxypeptidase D gene. This class of enzyme has been identified as a regulatory B-type metallocarboxypeptidase. Dysregulation of this protein may contribute to cancer disease progression, which is the case in breast cancer (due to the upregulation of the hormones 17β-estradiol and prolactin increase with the consequent rise in the expression in carboxypeptidase D), prostate cancer (via increasing levels of testosterone), hematopoietic cancer and several lymphomas [36]. Figure 6B shows the SE found in chr4 q31.1, which is co-localized with LINC00499, which may advocate for a high level of SE dynamics regulation through lncRNAs. Figure 6C depicts the p12.3 region of chr3, which has been linked with rare mutations to the onset of autoimmune DiGeorge-like syndrome through miRNA [37] and markers for advanced nasopharyngeal carcinoma prognosis [38]. The SE is co-localized with the ROBO1 gene, which encodes a member of the immunoglobulin gene superfamily—an integral membrane protein that coordinates axon guidance and neuronal precursor cell migration. This receptor is activated by SLIT-family proteins, SLIT and Robo1 expression is a biomarker for tumor progression in glioma patients [39]. Another HEK293 SE (Figure 6D) is found on the chromosome 2, p25.1 region and it is co-localized with the GRHL gene, encoding TF—a member of the grainyhead family. The product regulates DSG1 in the context of hair anchorage and epidermal differentiation, and it participates in the maintenance of the skin barrier. GRHL family members in general are a critical conduit for modulating the molecular landscape that guides cellular decision-making processes during proliferation, epithelial–mesenchymal transition (EMT) and migration [40]. The final of the top-five HEK293 super-enhancers is SE, which is located on the chr5 q23.2 (Figure 6E) and is co-localized with the MEGF-10 gene. Its product mediates cell proliferation and differentiation signaling [41] and is a critical mediator of apoptotic cell phagocytosis as well as amyloid-beta peptide uptake in the brain. In neuroblastoma cell lines, cell engulfment and adhesion factor gene MEGF10 is epigenetically repressed by DNA hypermethylation or by H3K27/K9 methylation [42], linking the complex regulation of SE intersecting regions. It was also shown that several members of the MEGF subfamily of orphan adhesion G-protein-coupled receptors (8 of 12) are preferentially expressed in pancreatic cancer stem cells and being stem-cell-unique, super-enhancer-associated genes had a higher probability of dropping out in the CRISPR screen in stem cell vs. non-stem cell conditions.

**Figure 7:**
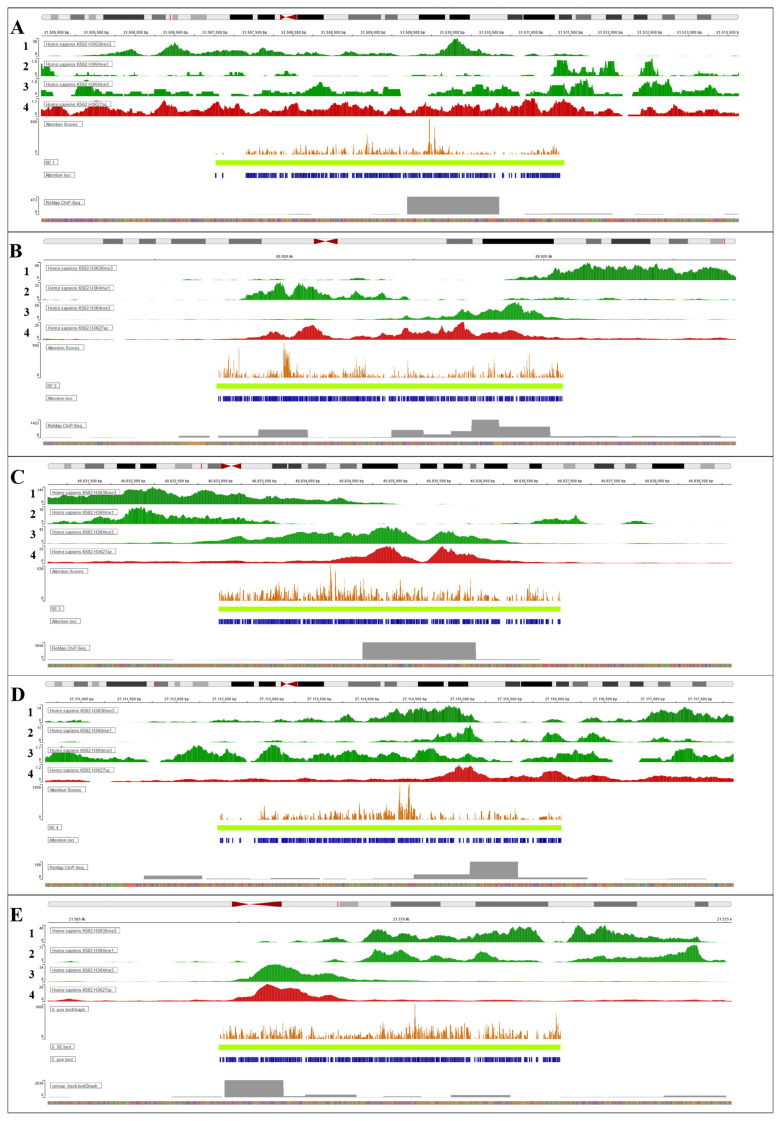
Visualization of intersection between epigenetic marks (dark green and red) and model attention positive class subsequences (blue) for 5 SEs (**A**–**E**; light green) from the K562 cell line. Epigenetic marks are numerated as follows: 1. H3K36me3, 2. H3K4me1, 3. H3K4me3, 4. H3K27ac. Values of attention score for each positive class subsequence represented as diagram (orange). ReMap Atlas track of regulatory regions is shown as a gray distribution pattern and is based on a comprehensive integrative analysis of all publicly available ChIP-seq data. Visualized in Integrative Genomics Viewer.

As can be seen from the super-enhancer regions listed for both cell lines, the algorithm is able to find super-enhancers along the entire length of the autosomes with high SE-positive attention scores. Thus, both HEK293 and K562 cells contained each, among others, two super-enhancers in the pericentromeric space (Figure 6A,C and Figure 7C,D) as well as one super-enhancer in the distal regions closer to the telomeres (Figure 6D and Figure 7B). It seems that the distribution of the model attention score values roughly reflects the cumulative (combined) effect of acetylation and trimethylation mark (H3K37a and H3K4me3) in each sample; only in some cases, it is not quite coinciding with the peak summits of the specified tracks. This result might be interesting because our model, which basically does not use any knowledge of epigenetic labels and has received only DNA sequence data as input, forms the integrative attention score value in such a manner as if it could describe some combined effect of a group of epigenetic labels; i.e., it also potentially includes information about some auxiliary regulatory label (e.g., from the so-called ‘broad epigenetic domain’ [32]), which we had not yet taken into account in the analysis.

The co-occurrence of H3K4me3 and H3K27ac modifications at enhancer elements of SEs suggests two possible scenarios of gene regulation. Either of these marks can be switched and perform expression regulation solely or work synergistically and provide a regulatory environment that supports the activation of genes essential during early development. For example, during the transition from pluripotency to differentiation in zebrafish (*Danio rerio*), particular enhancers were enriched for both H3K4me3 and H3K27ac marks, revealing them as functional in driving developmental genes expression [43].

In contrast, the K562 cell line showed interesting concordance between ChIP-Atlas data and evaluation metrics peaks (Figure 7). For example, the super-enhancer represented in Figure 7B showed relatively high peaks of epigenetic context aligned with the model’s evaluation metric peaks, suggesting a strong correlation between the model’s importance scores and the epigenetic marks in this cell line. In particular, the distribution of H3K4me3, H3K4me1, and H3K27ac within the locus is a sign of a highly active chromatin state associated with subsequences through nucleotide context solely. On the other hand, there is a case of negative intersection (Figure 7A), where we showed a weak association with all epigenetic marks within the locus except for a light significance of the H3K36me3 signal. H3K36me3 is commonly found in gene-coding regions and can act as a downregulation mark for promoters and enhancers, which aligns with the logic of a suppressed enhancer element within this particular super-enhancer. Figure 7A demonstrates the SE from the chr6 p21.32 locus. This SE is co-localized with the DDX39B gene, and its product plays pivotal roles in mRNA binding, splicing and export. This gene encodes a member of the DEAD box family of RNA-dependent ATPases mediating ATP hydrolysis during pre-mRNA splicing. Its upregulation was found in many tumors, including colorectal cancer, where DDX39B acts as an oncogene, directly binding to CDK6/CCND1 [44]. The DDX39B gene belongs to a cluster of genes localized in the vicinity of the genes encoding tumor necrosis factor alpha and tumor necrosis factor beta; all together, they are located within the human MHC class III region [45]. Thus, SE regulation on this gene may possibly have effects on the surrounding gene expression levels. The SE in Figure 7B is a part of the chr16 q24.3 region and is co-localized with the PIEZO1 gene. The protein encoded by this gene is a mechanotransduction ion channel. In the human body, PIEZO1 is widely distributed in the skin, bladder, kidney, lung, endothelial and bone-related cells [46]. Piezo1 was confirmed to control vascular (VSMC foam) cells transition through epigenetic changes, including a downregulation of the repressive H3K9me3 mark, leading to altered lipid metabolism and atherogenic gene expression [47]. Another SE from the top-five group of SE elements found in K562 was the one located on the chr5 p13.1 (Figure 7C). Within the calculated region of the SE was found the ribosomal RPL37 gene, which encodes a protein component of the 60S subunit. Autophagy-related RPL37 is believed to be an effector gene of miR-4516, whose upregulation is observed in lung cancer cells [48], highlighting the possible regulation of the surrounding SE distribution by ncRNA. Figure 7D shows the K562 SE from the chr6 p22.1 locus. Within this SE sequence, there are three co-localized genes: two histone genes, H2BC12 and H2AC12, and a microRNA gene—MIR3143. The differentially upregulated expression of H2BC12 is a promising biomarker for the diagnosis and prognosis of glioma (grades II and III) patients [49]; H2AC12 might also be soon regarded as a novel marker for cancer prognosis and aging [50]. Interestingly, the locus is not rich in activating histone marks, which is probably due to the changed epigenetic status of a terminally differentiated cell line. The SE from the chr22 q11.2 closes the top-five SEs calculated by GENA-LM (Figure 7E). The SE area covers two transcripts of GGT2, which has been lately regarded as a processed pseudogene [51]. GGT2 belongs to the gamma-glutamyltransferase (GGT; EC 2.3.2.2) gene family; it is a membrane-bound extracellular enzyme that cleaves gamma-glutamyl residue, being key to glutathione homeostasis and glutathione synthesis.

Possible differences in peak intersection might be also due to the different genomes assemblies used for ReMap visualizations, as the latest (4th) release of ReMap in 2022 presents uses mapping to the GRCh38/hg38 human assembly. It should also be noted that according to the intersection of the generalized peaks of the ReMap data (binding sites of transcriptional regulators) and the super-enhancer sequences we predicted, in most cases, the TF binding sites are located within the predicted super-enhancer locus.

One of the directions for future work involves implementing k-fold cross-validation to obtain a more robust estimate of model performance and further assess overfitting risks. While the high computational cost was a limiting factor in the current study, optimizing cross-validation strategies could enhance the generalizability and reliability of transformer-based predictions.

Although our study provides preliminary insights into gene regions potentially influenced by epigenetic dynamics, a more detailed analysis of the identified sequence motifs and their functional impact would require experimental validation. As a next step, we are designing experiments using CRISPR/Cas epigenetic editors to validate the regions of interest identified by the algorithm and to functionally characterize the associated phenotypes. These efforts will help refine the biological interpretation of the model’s predictions and assess their relevance in a broader genomic context.

Overall, the GENA-LM deep learning model was utilized to identify super-enhancers in HEK293 and K562 cell lines, revealing distinct patterns of epigenetic marks associated with gene regulation. We highlighted that H3K4me3 and H3K27ac were prevalent in HEK293 super-enhancers, suggesting potential roles in gene activation during early development despite a weak concordance of model significance scores with generic ChIP-Atlas peaks. In contrast, the K562 cell line results demonstrated a strong association between the model’s importance scores and epigenetic marks, indicating a highly active chromatin state. These findings reveal the effective integration of deep learning models with epigenetic data enhancing our understanding of SE functionality and gene expression regulation across different cell types.

## 4. Conclusions

Classifying super-enhancers and typical enhancers only by DNA sequence is still quite an ambitious task [52,53,54]. Challenges include insufficient data for many cell types, different cell annotation techniques (e.g., unsupervised clustering and supervised annotation), the tissue specificity of super-enhancers, and their large length, as well as the natural complexity of super-enhancers being a superposition of typical enhancer elements, which is a limitation of many models used in the field of sequence analysis.

Apart from that, the choice of a feature-encoding method in neural networks with robust representation power will have a solid impact on the accuracy of prediction of SEs based on DNA sequence hidden information exploitation.

Two models have been previously published that solve similar classification problem, namely the classification of SE and TE from sequence data only: DeepSE [27] and SENet [11]. The results of our study are comparable with previously obtained ones, and for some cell lines, for example, HEK293, they are superior to them in terms of the metrics used (Table 2).

In this study, we demonstrated the robustness of the GENA-LM deep learning model in classifying enhancers and super-enhancers using only hg38 genome nucleotide con-text without any prior knowledge of epigenetic labels.

To overcome the disadvantage of previously published algorithms—sequence length limitation, we used the BigBird model, which allowed us to consider sequences with length up to 36,000 bp [9]. Thanks to this, it was not necessary to filter super-enhancers by length, as in the case of the previously published SENet, where the limit was a length of 3000 bp [11], while SEs are usually more extensive [1,2]. This made it possible to collect data for a fine-tuning model, pre-trained on genomic data [9], for individual human cell lines. Additionally, we preprocessed the data for TE by adding flanking sequences to avoid data leakage due to the length difference between SE and TE.

Our results, based on the obtained evaluation metrics, indicate that the model effectively identifies super-enhancers across various cell lines, revealing epigenetic landscapes associated with gene regulation.

In the HEK293 cell line, we observed that super-enhancers frequently co-localize with H3K4me3 and H3K27ac marks, suggesting a potential role in gene activation and early developmental processes. These marks may also facilitate a regulatory environment sup-porting gene expression, despite a weak concordance between model significance scores and ChIP-Atlas peaks. On the other hand, analysis of the K562 cell line demonstrated a strong concordance between the importance scores and the epigenetic data, highlighting a highly active chromatin state within studied regions.

Therefore, fine-tuned GENA-LM not only captured the nucleotide context but may also inferred functional contextual dependencies associated with these epigenetic marks. The identified super-enhancers in K562 were enriched with H3K4me3, H3K4me1, and H3K27ac, which play important roles in maintaining the active chromatin states essential for dynamic gene expression regulation.

Furthermore, the presence in the calculated super-enhancer regions and concordant clustering of transcription factor binding sites (TFBSs) are strong predictors of local enhancer activity. According to our analysis, these sequence motifs were enriched in regions with high attention scores. Further fine tuning of the model to recognize these TFBS motifs, followed by in vitro validation, could improve its ability to classify and detect super-enhancers.

The sequence content of super-enhancers comprising long and short enhancer elements can influence their activity and specificity. This relationship can be explored further by analyzing the sequence characteristics of regions with high attention scores obtained by our adaptation of the GENA-LM. Thus, the higher DNA sequence GC-content is correlated with enhancer activity. By incorporating GC content as a feature in the model, the SE classification accuracy might be enhanced.

Another extension to the current algorithm would be expanding the training and test datasets with more diverse malignant cell OMICS profiles. Enhanced epigenomic and transcriptomic profiling in different cell states (e.g., MEL and MES subtypes) can result in divergent phenotypes. Understanding these profiles in the context of attention scores can provide insights into cell-specific enhancer activity.

Overall, our analysis reinforces the potential of deep learning models in enhancing our understanding of super-enhancer functionality and their impact on gene expression. By associating predicted super-enhancers with various epigenetic marks, we revealed dependencies that may play critical roles in cellular development and malignization.

Moving forward, a range of emerging technologies—such as advanced magnetic nanotags [55], label-free biosensors [56,57,58], and cloud-based genome-wide data analysis [59]—presents new opportunities to refine our understanding of SE biology, particularly in oncological contexts. These tools may reshape current conceptual frameworks for super-enhancers and pave the way for disease-specific breakthroughs.

## Figures and Tables

**Table 1:** The datasets used for the fine tuning.

Cell Line	Number of Sequences	Resources
HeLa	3150	dbSuper, SEdb, Encode
HEK293	1260	SEdb, EnhancerAtlas 2.0
H2171	872	dbSuper, SEdb, EnhancerAtlas 2.0
Jurkat	2374	dbSuper, SEdb, EnhancerAtlas 2.0
K562	4016	dbSuper, SEdb, EnhancerAtlas 2.0
MM1S	1729	dbSuper, SEdb, Encode
U87	2626	dbSuper, SEdb, EnhancerAtlas 2.0

**Table 2:** Performance comparison of fine-tuned GENA-LM on different human cell lines datasets.

Cell Line	Accuracy	F1	Precision	Recall	ROC AUC	MCC
HeLa	0.5978	0.5973	0.5988	0.5984	0.5978	0.1966
HEK293	0.8093	0.8096	0.8098	0.8095	0.8093	0.6180
H2171	0.7461	0.7478	0.7489	0.7487	0.7461	0.4952
Jurkat	0.7008	0.6842	0.7352	0.6947	0.70008	0.4317
K562	0.7519	0.7491	0.7569	0.7500	0.7519	0.5075
MM1S	0.7557	0.7575	0.7658	0.7601	0.7557	0.5230
U87	0.6521	0.6522	0.6523	0.6521	0.6514	0.3026
Integrative	0.6944	0.6963	0.6986	0.6980	0.6944	0.3933

**Table 3:** Performance of the integrative model on the datasets for specific cell lines.

Cell Line	Accuracy	F1	Precision	Recall	ROC AUC	MCC
HeLa	0.5698	0.5555	0.5802	0.5698	0.5698	0.1496
HEK293	0.6696	0.6800	0.6853	0.6865	0.6696	0.3538
H2171	0.5752	0.5541	0.5904	0.5721	0.5752	0.1642
Jurkat	0.6610	0.6612	0.6616	0.6611	0.6610	0.3218
K562	0.4684	0.4559	0.4642	0.4768	0.4684	−0.0687
MM1S	0.6062	0.5981	0.6231	0.6127	0.6062	0.2297
U87	0.5698	0.5555	0.5802	0.5698	0.5698	0.1496

## Data Availability

Data are contained within the article.

## Supplementary Materials

The following supporting information can be downloaded at https://www.mdpi.com/article/10.3390/biology14020172/s1, Table S1: The hyperparameters for GENA-LM fine tuning; Table S2: Evaluation metrics used to access the performance of the pretrained GENA-LM; Table S3: McNemar’s test results for accuracy value (H0: GENA-LM performs like a random classifier, H1: GENA-LM does not perform like a random classifier); Table S4: Kolmogorov–Smirnov test results for the comparation of GENA-LM with random classifier on random sequences from human genome (H0: GENA-LM predictions are distributed like predictions of a random classifier, H1: GENA-LM predictions are not distributed like predictions of a random classifier); Figure S1: Gena-LM architecture; Figure S2: Changing of evaluation metrics during training; Figure S3: The t-SNE visualization of the features distribution for the GENA-LM transformer layer output; Figure S4: The UMAP visualization of the features distribution for the GENA-LM transformer layer output.
