# Supplementary material for: Sequence-Only Prediction of Super-Enhancers in Human Cell Lines Using Transformer Models
Source: Biology (Basel). 2025 Feb 7;14(2):172. doi: 10.3390/biology14020172 (PMC11852244; doi:10.3390/biology14020172)
Supplement: Supplementary file 1 [file biology-14-00172-s001.zip › Supplementary.pdf]

Supplementary materials

Sequence-Only Prediction of Super-Enhancers in Human Cell Lines Using Transformer Models

Table S1. The hyperparameters for GENA-LM fine-tuning. The used loss function - cross-entropy loss.

Hyperparameter	Value
Number of training epochs for classifier fine-tuning	5
Number of training epochs for the full model fine-tuning	10
Learning rate for classifier fine-tuning	1.00E-04
Learning rate for the full fine-tuning	1.00E-05
Weight decay	0.01
Gradient accumulation steps	32
Per device train batch size	2
Per device evaluation batch size	16

Table S2. Evaluation metrics used to access the performance of the pretrained GENA-LM.

Metric	Definition
Accuracy	(TP + TN) / (TP+FN+FP+TN), where TP, TN, FP, and FN represent the number of true positives, true negatives, false positives, and false negatives, respectively
Balanced Accuracy	(sensitivity + specificity) / 2, where sensitivity = TP / (TP + FN) and specificity = TN / (TN + FP). As soon as classes in the binary classification are balanced, the accuracy equals the balanced accuracy
ROC AUC	the area under the Receiver Operating Characteristic curve, which is a plot of the true positive rate against the false positive rate at each threshold setting
Recall (Sensitivity)	TP / (TP + FN), which represents the proportion of true positives that are correctly identified
Precision	TP / (TP + FP), which represents the proportion of true positives among the predicted positives
F1-score	2 x [(precision x recall) / (precision + recall)], which is the harmonic mean of precision and recall
Matthews Correlation Coefficient (mcc)	(TP x TN - FP x FN) / √[(TP+FP)(TP+FN)(TN+FP)(TN+FN)] which considers true/false positives and true/false negatives.

Table S3. McNemar's test results for accuracy value (H0: GENA-LM performs like a random classifier, H1: GENA-LM does not perform like a random classifier).

Dataset	Cell line specific model		Integrative model	
	tets statistic	p-value	Test statistic	p-value
HeLa	5.3772	0.0204*	2.7433	0.0977
HEK293	17.9667	2.2481E-05**	5.9797	0.0145*
H2171	8.1290	0.0044**	0.7701	0.3802
Jurkat	15.5290	8.1249E-05**	10.3036	0.0013**
K562	40.5204	1.9458E-10**	0.7394	0.3899
MM1S	17.4401	2.9651E-05**	3.5651	0.0590
U87	10.0562	0.0015**	2.3020	0.1292
integrative	15.4372	8.5293E-05**	-	-

* p-value < 0.05

** p-value < 0.01

Table S4. Kolmogorov-Smirnov test results for the comparation of GENA-LM with random classifier on random sequences from human genome (H0: GENA-LM predictions are distributed like predictions of a random classifier, H1: GENA-LM predictions are not distributed like predictions of a random classifier).

Model fine-tuned on	tets statistic	p-value
HeLa data	0.15	0.9831
HEK293 data	0.31	0.1081
H2171 data	0.14	0.9150
Jurkat data	0.1	0.9999
K562 data	0.35	0.1746
MM1S data	0.1	0.9667
U87 data	0.2	0.8320
Integrative data	0.26	0.2536

See attached PDF-file "Figure S1.pdf"

Figure S1. Gena-LM architecture. The classification head includes the following: dropout, linear layer followed by NewGELUActivation, dropout, linear layer. The output shape is (1, 2) since the model solves the problem of binary classification. Fine-tuning was performed first for the classifier (5 epochs), and then for all layers of the model (10 more epochs).

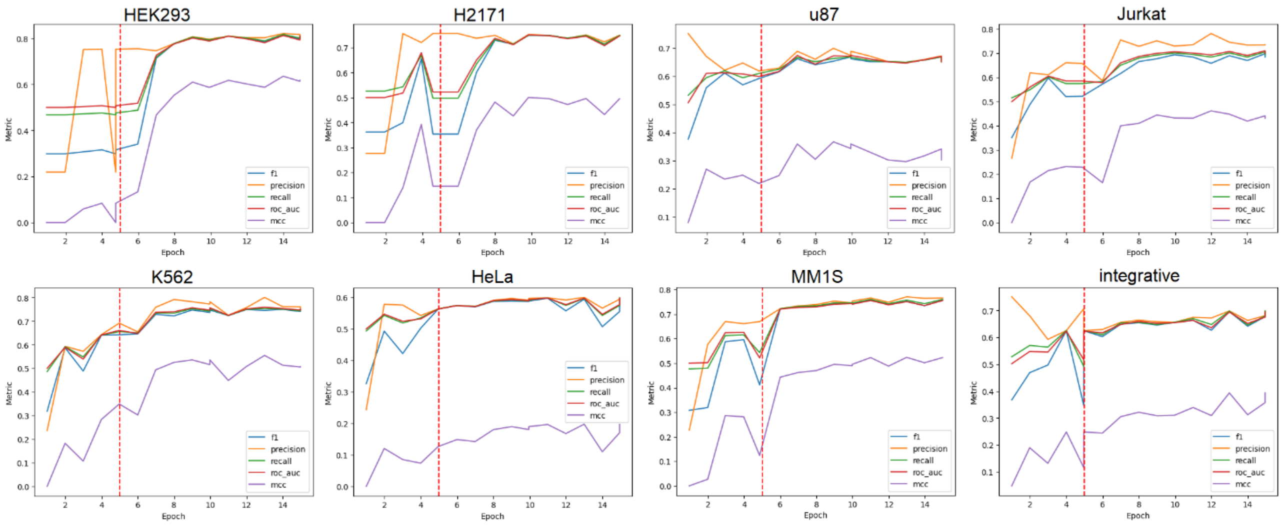

Figure S2. Changing of evaluation metrics during training. The red line represents the epoch when fine-tuning of all model layers begins.

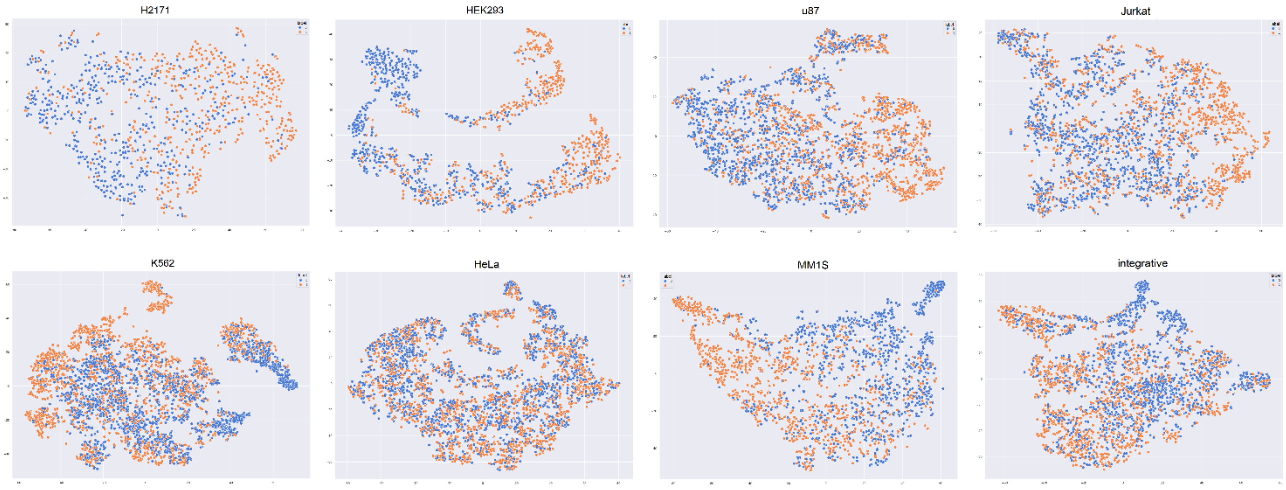

Figure S3. The t-SNE visualization of the features distribution for the GENA-LM transformer layer output.

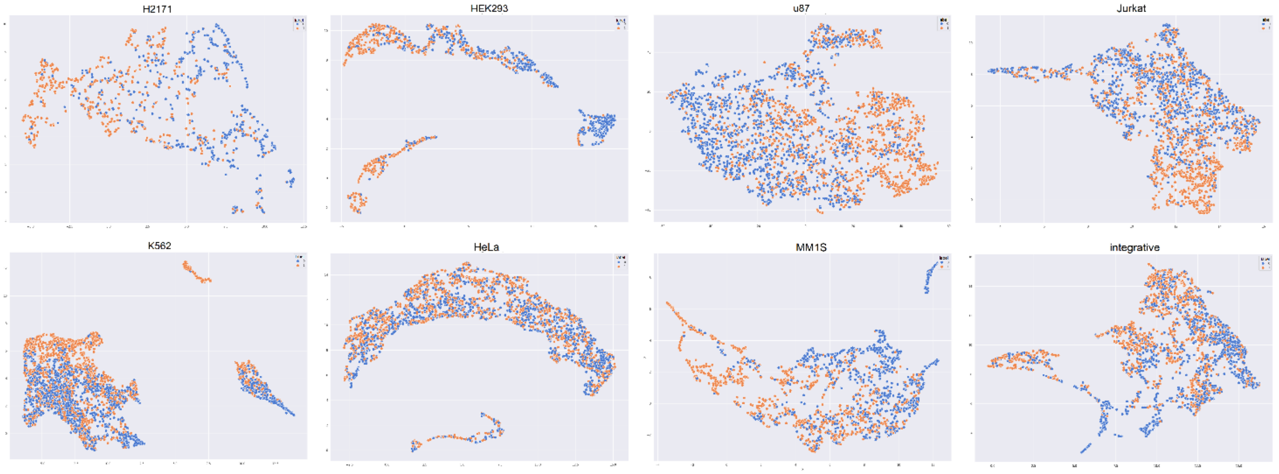

Figure S4. The UMAP visualization of the features distribution for the GENA-LM transformer layer output.
