# Supplementary figures and images for: Sequence-Only Prediction of Super-Enhancers in Human Cell Lines Using Transformer Models
Source: Biology (Basel). 2025 Feb 7;14(2):172. doi: 10.3390/biology14020172 (PMC11852244; doi:10.3390/biology14020172)

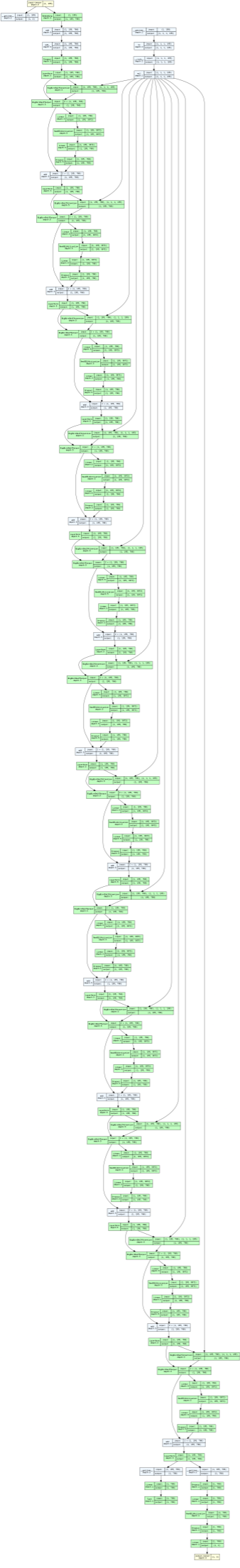

Figure S1. gene-in-bigbird-base-2l2 architecture

Supplement: Supplementary file 1 [file biology-14-00172-s001.zip › Figure S1.pdf]
